# Leaf Turgor Loss Does Not Coincide With Cell Plasmolysis in Drought‐Tolerant Chaparral Species

**DOI:** 10.1111/pce.15505

**Published:** 2025-03-27

**Authors:** Leonie C. Schönbeck, Carolyn Rasmussen, Louis S. Santiago

**Affiliations:** ^1^ Department of Botany & Plant Sciences University of California, Riverside Riverside California USA; ^2^ Southern Swedish Forest Research Center Swedish University of Agricultural Sciences Alnarp Sweden; ^3^ Smithsonian Tropical Research Institute Apartado Panama

**Keywords:** chaparral, drought, heat, histology, thermal tolerance, turgor loss point

## Abstract

The water potential at which leaf cells lose turgor (*Ψ*_TLP_) is a useful predictor of whole‐plant drought tolerance and biome wetness. However, many plants can achieve water potential values below *Ψ*_TLP_ and recover, raising questions about the physiological processes that occur below *Ψ*_TLP_. We established a controlled greenhouse experiment to induce turgor loss on six shrub species from a Mediterranean‐type ecosystem in Southern California and characterised physiological and leaf‐structural adjustments to *Ψ*_TLP_. We documented seasonal adjustments in *Ψ*_TLP_, both with and without applied drought. Stomatal closure always occurred below *Ψ*_TLP_, and the margin between the two phenomena increased with lower *Ψ*_TLP_. Drought tolerance was strongly correlated with heat tolerance. Most histological responses to *Ψ*_TLP_ involved shrinkage of both spongy mesophyll cells and intercellular air spaces, leading to reduced leaf thickness, but not plasmolysis. Overall, our results indicate a propensity to reach *Ψ* values far below *Ψ*_TLP_ and maintain function for extended periods of time in Southern California shrubs. Whereas species in many ecosystems fall below *Ψ*_TLP_ for brief periods of time, the erratic nature of precipitation patterns makes Southern California an outlier in the range of operational plant water potentials.

## Summary statement

Drought‐tolerant chaparral species can survive months with water potentials below their turgor loss point. We find that turgor loss point can vary with drought and temperature increase and does not coincide with stomatal closure or cell plasmolysis, as commonly assumed, in these species.

## 1 Introduction

Drought tolerance is a composite trait consisting of plant structural properties, as well as a set of morphological and biochemical adjustments (Santiago et al. 2016; Pivovaroff et al. 2016; Choat et al. 2018). Generally, leaf drought tolerance is a good indicator of whole‐plant drought tolerance and, on a global scale, is related to biome wetness (Bartlett et al. 2012b). Specifically, leaf turgor loss point (*Ψ*_TLP_), the water potential at which leaf cells lose their positive pressure and wilt, is an excellent predictor of drought tolerance across biomes (Tyree and Hammel 1972; Bartlett et al. 2012b). However, within any one biome or ecosystem, a plethora of different drought strategies exist besides low leaf *Ψ*_TLP_, potentially blurring the strong global relationship between *Ψ*_TLP_ and whole‐plant drought tolerance. For example, drought avoidance traits such as deep roots and drought‐deciduousness can increase plant survival during drought (Lopez‐Iglesias et al. 2014). The cuticle, a continuous lipophilic layer on the leaf epidermis, creates a barrier between the leaf and the atmosphere and reduces the rate of tissue water loss when stomata are closed to varying degrees across species (Duursma et al. 2019). A common leaf characteristic of shrub species growing in Mediterranean‐type climates is sclerophylly, a tough and leathery leaf form with lignified elements that provide structural support in periods of extreme water shortage (Schimper 1903; Seddon 1974; Edwards et al. 2000). In addition, leaf biochemical changes, such as osmotic adjustment, can alter *Ψ*_TLP_, by accumulation of osmotically active compounds such as sugars, proteins or lipids. Thus, whereas characterising *Ψ*_TLP_ is important for understanding drought tolerance, a variety of factors mediate when leaves of a particular species arrive at that point.

**Figure 1:**
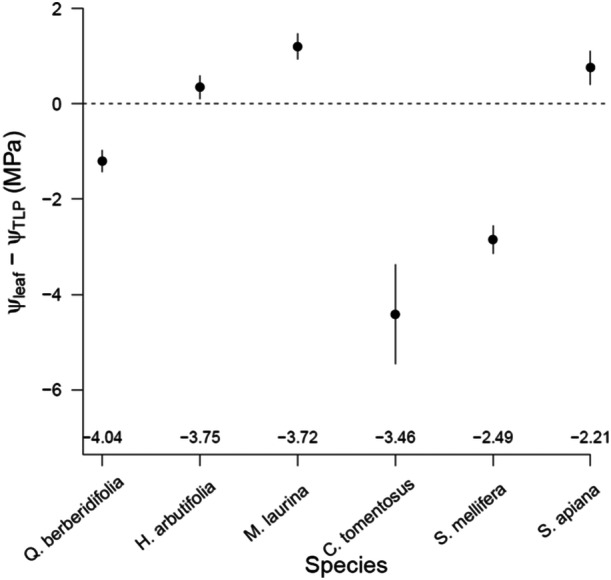
Difference between minimum *Ψ*_leaf_ at the end of the dry season (September 2021) and *Ψ*_TLP_ measured at the same time. Actual *Ψ*_TLP_ is shown in numbers above the x‐axis. Symbols indicate the *Ψ*_leaf_ − *Ψ*_TLP_. Negative values indicate *Ψ*_leaf_ was lower (more negative) than the *Ψ*_TLP_. Error bars show SE (*n* = 5). Measurements were taken at the Santa Margarita Ecological Reserve on five individuals of each species.

The (sub)cellular consequences of turgor loss and the relationship between *Ψ*_TLP_ and other drought‐tolerance parameters present a complex array of interacting factors (Brodribb et al. 2003; Bartlett et al. 2016; Scoffoni et al. 2023). Turgor loss is associated with leaf wilting and cell plasmolysis and is often seen as the step towards more dangerous structural damage. When the positive water pressure (turgor) that maintains cell structure is lost, for example, due to drought, cells plasmolyse or collapse, causing the leaf to wilt (Cochard et al. 2002). However, the exact safety margins that allow plants to survive periods below their *Ψ*_TLP_ differ among species. Plants in the unique Chaparral ecosystem in Southern California can survive months at several megapascals below *Ψ*_TLP_ without leaf mortality or branch dieback (Figure 1), raising questions about the physiological consequences of maintaining living tissue beyond turgor loss and the ultimate consequences for leaves of these extreme drought‐tolerant plants. The interaction between drought and heat adds an extra level to this question, as leaves regulate water use in response to both drought and temperatures. Photosynthesis is highly temperature dependent, and leaf temperatures exceeding the optimum result in reduced photosynthesis due to slower enzymatic processes, up to a point—generally at temperatures reaching 40°C or higher—where the photosystem becomes impaired (Berry and Bjorkman 1980). Evaporation from leaves, therefore, also regulates leaf temperatures. Yet, when leaf water potential approaches *Ψ*_TLP_, leaf cooling and water conservation represent an inevitable trade‐off. In line with balancing the need to maintain leaf water status and favourable leaf temperatures for photosynthesis, there is some data (Sastry et al. 2018; Mitchell 2021; Münchinger et al. 2023), and more theories (Smékalová et al. 2014), suggesting that drought and heat tolerance are related and exhibit cross‐tolerance such that drought‐tolerant plants also have a higher thermal limit.

The Mediterranean‐type ecosystem of Southern California has unique climatic and vegetation properties. With an abrupt interface between the relatively hot landmass and the cold ocean, and with storms directing precipitation systems directly from a large expanse of the Pacific Ocean, it lacks the moderating effect of other such regions, such as the Mediterranean Basin. For this reason, California has the most erratic precipitation pattern of any of the five Mediterranean‐type climate zones (Cowling et al. 2005), with its long, hot and dry summers punctuated by short mild winters at the mercy of mercurial weather patterns and sea surface temperature anomalies mediated by El Niño and La Niña events (Cook et al. 2009; Griffin and Anchukaitis 2014). Unpredictable rainfall patterns in Southern California may result in droughts for up to several years, exposing plants to some of the lowest water potentials (*Ψ*_leaf_) worldwide (Jacobsen et al. 2007; Pivovaroff et al. 2016). Human‐induced climatic changes have made these patterns even more dynamic, resulting in several recent mega‐droughts in 2014–2018 and 2020–2021 (Griffin and Anchukaitis 2014; Leeper et al. 2022) and a 2000‐year low rainfall in the period between 2020 and 2021 (Keeley and Syphard 2021).

We connected plant physiological processes with leaf cellular structure, to better understand the mechanisms and consequences of extended turgor loss in six native Californian Chaparral species. First, we tested the assumption that these species can reach *Ψ*_leaf_ far below their turgor loss without signs of mortality under field conditions. We also established a controlled greenhouse experiment to induce turgor loss and characterise adjustments to *Ψ*_TLP_, photosynthetic gas exchange, leaf temperature, thermal tolerance, plant water status, non‐structural carbohydrates and cell structure, of well‐watered and sub‐turgor plants. We hypothesised that: (1) chaparral and coastal sage scrub species have the capability to adjust their *Ψ*_TLP_ in response to drought, (2) *Ψ*_TLP_ and *T*_50_ are correlated such that high drought tolerance translates to high heat tolerance and (3) leaf *Ψ*_TLP_ results in leaf shrinkage and selective cell plasmolysis while conserving photosynthetic function.

## 2 Methods

### 2.1 Species

Six shrub species from chaparral (*Ceanothus tomentosus*, *Heteromeles arbutifolia*, *Malosma laurina* and *Quercus berberidifolia*) and coastal sage scrub (*Salvia apiana* and *Salvia mellifera*) vegetation types were selected based on estimated rooting depth and *Ψ*_TLP_, with the aim to select species along a wide range of both indicators (Table S1). Six individuals of each species were planted in 18‐L pots with a mix of organic soil (bark, plaster sand and peat moss 1:2:1) and perlite (in a 1:4 perlite:soil mixture), to create a well‐draining soil. The plants were put in a greenhouse where temperature and humidity were monitored at 30‐min intervals (Figure S1).

### 2.2 Drought Experiment

In May 2022, three plants of each species were exposed to drought treatment, while the other three plants were kept well‐watered. Watering was done 3 times per week. Well‐watered plants were watered to soil saturation. The amount of water given to the drought‐stressed plants was determined by the *Ψ*_leaf_ of each individual, measured weekly on one leaf per plant (see below; Figure S2). The target was a gradual decrease in *Ψ*_leaf_ until below the determined *Ψ*_TLP_ for each species. *Ψ*_TLP_ was measured before the treatment, in April 2022, and again at the end of the treatment period, in July 2022, to confirm plant water status was indeed reaching below the reference *Ψ*_TLP_ (Schönbeck et al. 2023). In addition, soil volumetric water content (VWC, %) was measured weekly.

### 2.3 Turgor Loss Point and Water Potential

*Ψ*_TLP_ was measured using a vapour pressure osmometer (VAPRO 5600, Wescor, Logan, Utah) (Bartlett et al. 2012a). One leaf per plant was collected and rehydrated overnight with the petiole submerged in water. An 8 mm Ø leaf disc was cut centrally between the leaf midrib and margin and tightly wrapped in aluminium foil. Veins were avoided as much as possible. Discs were submerged in liquid N for 2 min. After unwrapping, the disc was punctured 10–15 times with a sharp pin and immediately sealed in the osmometer chamber (VAPRO 5600, Wescor, Logan, Utah). The leaf was exposed to the air for < 40 s during the entire process from cutting to enclosing in the chamber, minimising the chance of evaporation before measurement. The repeated‐measurements mode was used to take 10 measurements at an interval of ± 2 min. This was sufficient to reach an equilibrium indicated by a < 5 mmol difference between two consecutive measurements. The measurements were plotted over time and checked for outliers and a flattening curve. Then, the lowest value reached, generally the last measurement in the sequence, was used as a final value. *Ψ*_TLP_ was calculated using the linear regression equation provided by Bartlett et al. (2012a):

$$\pi_{\mathrm{tlp}}=0.832\pi_{\mathrm{osm}}-0.631$$

This formula is based on a wide range of species around the globe and is assumed to give a generally good fit of *Ψ*_TLP_. We compared the obtained values with those found in literature based on pressure–volume curves to eliminate the possibilities for artefacts using the osmometer method or from overnight rehydration (Table S1; Figure S3) (Bowman and Roberts 1985; Davis and Mooney 1986; Pivovaroff et al. 2016; Abate et al. 2021). While overhydration would result in an underestimated *Ψ*_TLP_ by the osmometer method, we conclude from these tests that our samples are on or even below reported values from the literature and that we can exclude the possibility of overhydration.

Once per week, one leaf per plant was collected between noon and 2 PM and measured for midday *Ψ*_leaf_ with the Scholander pressure chamber (PMS Instruments, Albany, Oregon) (Figure S2). These values determined the amount of water given. The plants had sufficient leaves to ensure leaf removal did not have a significant effect on carbon or hydraulic status.

To create Figure 1, *Ψ*_TLP_ and minimum *Ψ*_leaf_ were determined in field conditions at the Santa Margarita Ecological Reserve (33°29′ N, 117°09′ W). Minimum *Ψ*_leaf_ was measured mid‐September 2021, after a dry summer, approximately 1 week before the first occurrence of seasonal rain. Five individuals per species and one leaf per individual were measured using the methods described above.

### 2.4 Thermal Tolerance

In April and July 2022, before and towards the end of the drought treatment, leaf heat tolerance (*T*_50_) was determined for the six species. *T*_50_ was assessed using a temperature assay of the maximum quantum yield of PSII (*F*_*v*_/*F*_*m*_), following Curtis et al. (2014). A 1 cm Ø leaf disc was cut centrally between the leaf midrib and margin where possible and sealed in a Whirl‐Pak bag (Whirl‐Pak Filtration Group, Chicago, Illinois, the United States). The Whirl‐Pak bags with leaf discs were submerged in a hot‐water bath at a series of temperatures—25, 30, 35, 38, 42, 46, 50, 54, 58 and 60°C—for 15 min. Earlier experiments showed that leaf discs reach water temperature within 2 min (Mitchell 2021). The leaf discs were then dark‐adapted with leaf clips for 20 min before measuring photosynthetic yield (*F*_*v*_/*F*_*m*_) with a MINI‐PAM fluorometer (Walz GmbH, Effeltrich, Germany). The *T*_50_ was calculated as the temperature at which *F*_*v*_ */F*_*m*_ had declined by 50% of the maximum, using a Weibull function to fit *F*_*v*_/*F*_*m*_ to temperature (fitplc package in R software) (Duursma and Choat 2017).

### 2.5 Gas Exchange

Photosynthesis (*A*, µmol m^−2^ s^−1^), stomatal conductance (*g_s_*, mol m^−2^ s^−1^) and transpiration (*E*, mol m^−2^ s^−1^) were measured biweekly starting the week before treatments commenced, in April 2022, with a LiCor LI‐6400 system (LiCor Inc., Lincoln, Nebraska, the United States) (Figure S4). One leaf per plant was clipped in the cuvette, set to 400 ppm CO_2_, 28°C, relative humidity of 60%–70%, photosynthetic active radiation (PAR) of 1200 µmol m^−2^ s^−1^ and flow of 500 µmol m^−2^ s^−1^. The leaf was left acclimating for at least 15 min and until stable *A* and *g_s_*. Three measurements were logged within 30 s. The three measurements were averaged during post‐processing. Intrinsic water use efficiency (iWUE) was calculated with *A*/*g_s_* (µmol/mol).

To calculate *Ψ*_gs90_ (stomatal closure), linear models were fitted with *Ψ*_leaf_ as the predictor and ln(*g_s_*) as the dependent variable. Data from well‐watered and dry plants were pooled to get the full range from *g*_smax_ (maximum measured *g_s_* value in well‐watered plants) to stomatal closure. The equation was then solved to calculate *Ψ*_leaf_ at 10% of *g*_smax_.

### 2.6 Non‐Structural Carbohydrates

Leaf material was collected in April and July 2022, on the same days as a collection for *Ψ*_TLP_ and thermal tolerance. The leaves were dried at 60°C until stable weight was achieved and then ground to a fine powder. NSCs were analysed following the protocol as described in Wong (1990) adapted according to Hoch et al. (2002). NSCs are defined here as low molecular weight sugars (glucose, fructose and sucrose) plus starch. 8–10 mg of ground material was boiled in 2 mL distilled water for 30 min. After centrifugation, an aliquot of 200 µL was treated with Invertase and Isomerase from baker's yeast (Sigma‐Aldrich, St. Louis, Missouri, the United States) to degrade sucrose and convert fructose into glucose. The total amount of glucose (sugars) was determined photometrically at 340 nm in a 96‐well microplate photometer (HR 7000, Hamilton, Reno, Nevada, the United States) after enzymatic conversion to gluconate‐6‐phosphate (hexokinase reaction, hexokinase from Sigma Diagnostics, St. Louis, Missouri, the United States). The total amount of NSC was measured by taking 500 µL of the extract (including sugars and starch) incubated with a fungal amyloglucosidase from *Aspergillus niger* (Sigma‐Aldrich, St. Louis, Missouri, the United States) for 15 h at 49°C to digest starch into glucose. Total glucose (corresponding to NSC) was determined photometrically as described above. The concentration of starch was calculated as NSC minus free sugars. Pure starch and glucose, fructose and sucrose solutions were used as standards and standard plant powder (Orchard leaves, Leco, St. Joseph, Michigan, the United States) was included to control reproducibility of the extraction. NSC concentrations are expressed on a per cent dry matter basis. Because all samples were run in a single laboratory with no change in protocol during the laboratory processing of samples, issues with comparison of results across methods or labs were obviated (Quentin et al. 2015).

### 2.7 Light Microscopy

Several leaf discs of 0.5 cm Ø were cut from the middle section of the leaves, between the mid‐vein and the edge. Leaf discs were immediately put in a 2%:2% formaldehyde:glutaraldehyde solution in 200 mM cacodylate buffer (Ruzin 1999). The fixation solution was refreshed after 4 h and leaves were kept under refrigeration until further use.

The leaf discs were gradually dehydrated in a series of increasing ethanol concentrations. They were gradually infiltrated with Spurr's resin, first overnight in a 50:50 (v/v) resin:ethanol solution and then overnight in 100% resin. Lastly, they were embedded in 100% resin in silicone moulds for > 48 h in an oven at 70°C. Samples were sectioned in the transverse plane at 0.5–1 µm thickness (depending on the ease of cutting and the clarity of the sections per species) with a glass knife (LKB Instruments) on a rotary microtome (Leica Lietz DMRB; Leica Microsystems) and stained with 0.01% toluidine blue in 1% sodium borate (w/v). Slides were imaged with a 4× or 10× objective using a light microscope (Leica Lietz DMRB; Leica Microsystems) and a camera utilising Amscope Imaging (Amscope). The following measurements were taken: the thickness of leaf, cuticle, epidermal cells, palisade mesophyll, spongy mesophyll, abaxial epidermal cells and the two‐dimensional area of intercellular space in the spongy mesophyll. All measurements were then standardised to the measured leaf thickness and presented in percentages.

### 2.8 Statistical Analyses

The difference in *Ψ*_TLP_ between drought and well‐watered plants was tested in a two‐way ANOVA with species and treatment as fixed factors. Separate mixed effects models were run for each species to test the effect of time and treatment on the absolute value of *Ψ*_TLP_. The individual was included as a random effect to correct for repeated measurements.

Treatment differences of cell structural traits were done using a two‐way ANOVA where the variable was tested against species and treatment. In case of significant interactions, a Tukey HSD test was done to study the specific group differences. Another two‐way ANOVA was carried out to test the treatment effect between evergreen and deciduous grouped species. The normality of the residuals and homogeneity of variance were confirmed.

Correlation analyses were carried out initially for the parameters of interest: *Ψ*_TLP_ versus *T*_50_, leaf mass per area (LMA), *A*, *g_s_*, iWUE (*A*/*g_s_*) and gs90. Then, a full correlation analysis was run with all physiological and histological parameters to detect any correlations that were not subject to a specific hypothesis. Significant correlations of interest were plotted in an x–y graph without any assumption of causal relationship.

All statistical analyses were done in R version 4.3.1 (R Core Team 2015).

## 3 Results

### 3.1 Species' Plasticity of *Ψ*_TLP_

**Figure 2:**
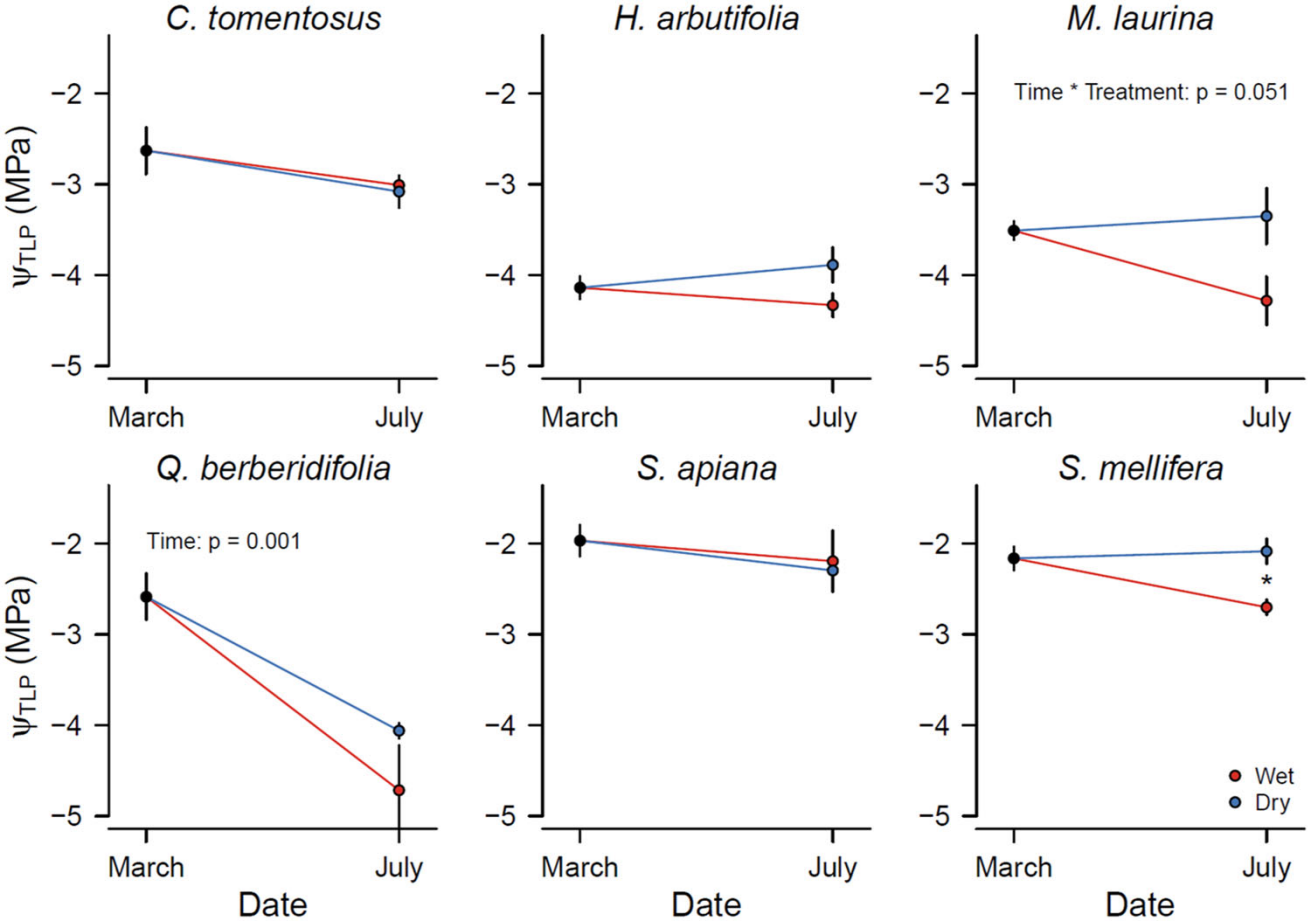
Turgor loss point (*Ψ*_TLP_) as measured in March and July 2022. The value in March shows the average of all plants together. The values in July distinguish between well‐watered (blue) and drought‐stressed (red) plants. Average ± SE are shown. Asterisks indicate significant species differences in July.

The deciduous *S. mellifera* and *S. apiana* had the least negative *Ψ*_TLP_ of all species (−2.2 and −2 MPa resp. at the beginning of the greenhouse drought experiment). *C. tomentosus* and *Q. berberidifolia* followed, while the lowest *Ψ*_TLP_ were found in *Heteromeles arbutifolia* and *Mangifera laurina* (−4.1 and −3.6 MPa resp.) (Figure 2). On average, drought‐stressed plants had lower *Ψ*_TLP_ than well‐watered plants (Δ*Ψ*_TLP_ = 0.41 ± 0.14 MPa for all species pooled, *p* < 0.01). In *Q. berberidifolia*, both well‐watered and drought‐stressed plants reduced *Ψ*_TLP_ significantly between March and July, indicating a seasonal change that was partly independent of soil moisture (Figure 2, Table S2).

### 3.2 Non‐Structural Carbohydrates

NSC levels in the leaves decreased with drought in *C. tomentosus*, *H. arbutifolia*, *M. laurina* and *Q. berberidifolia*, but increased in the two *Salvia* species (Figure S5, Table S3). While the decrease in the evergreen species was mainly caused by reductions in starch content, the increase in NSC in *Salvia* was mainly caused by a steep increase in sugar concentration. Sugar concentrations were not correlated with *Ψ*_TLP_.

### 3.3 Relation Between Turgor Loss and Other Physiological Traits

**Figure 3:**
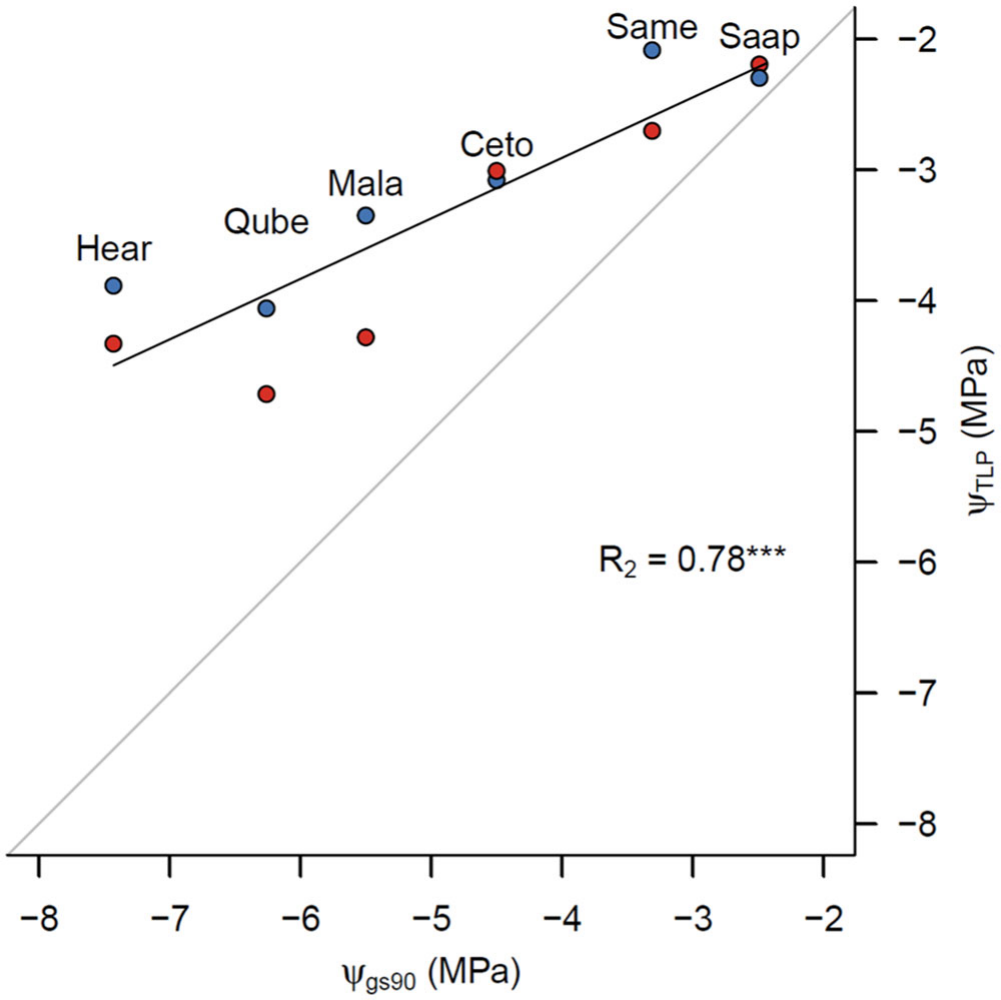
The relationship between turgor loss point (*Ψ*_TLP_) and gs90. Blue symbols indicate *Ψ*_TLP_ in well‐watered plants, and red symbols are drought‐stressed plants. The light‐grey line indicates a 1:1 line, showing a hypothetical simultaneous *Ψ*_TLP_ and gs90. The black line shows a linear regression. Abbreviations: Hear: *H. arbutifolia*, Qube: *Q. berberidifolia*, Mala: *M. laurina*, Ceto: *C. tomentosus*, Same: *S. mellifera*, Saap: *S. apiana*. [Color figure can be viewed at wileyonlinelibrary.com]

**Figure 4:**
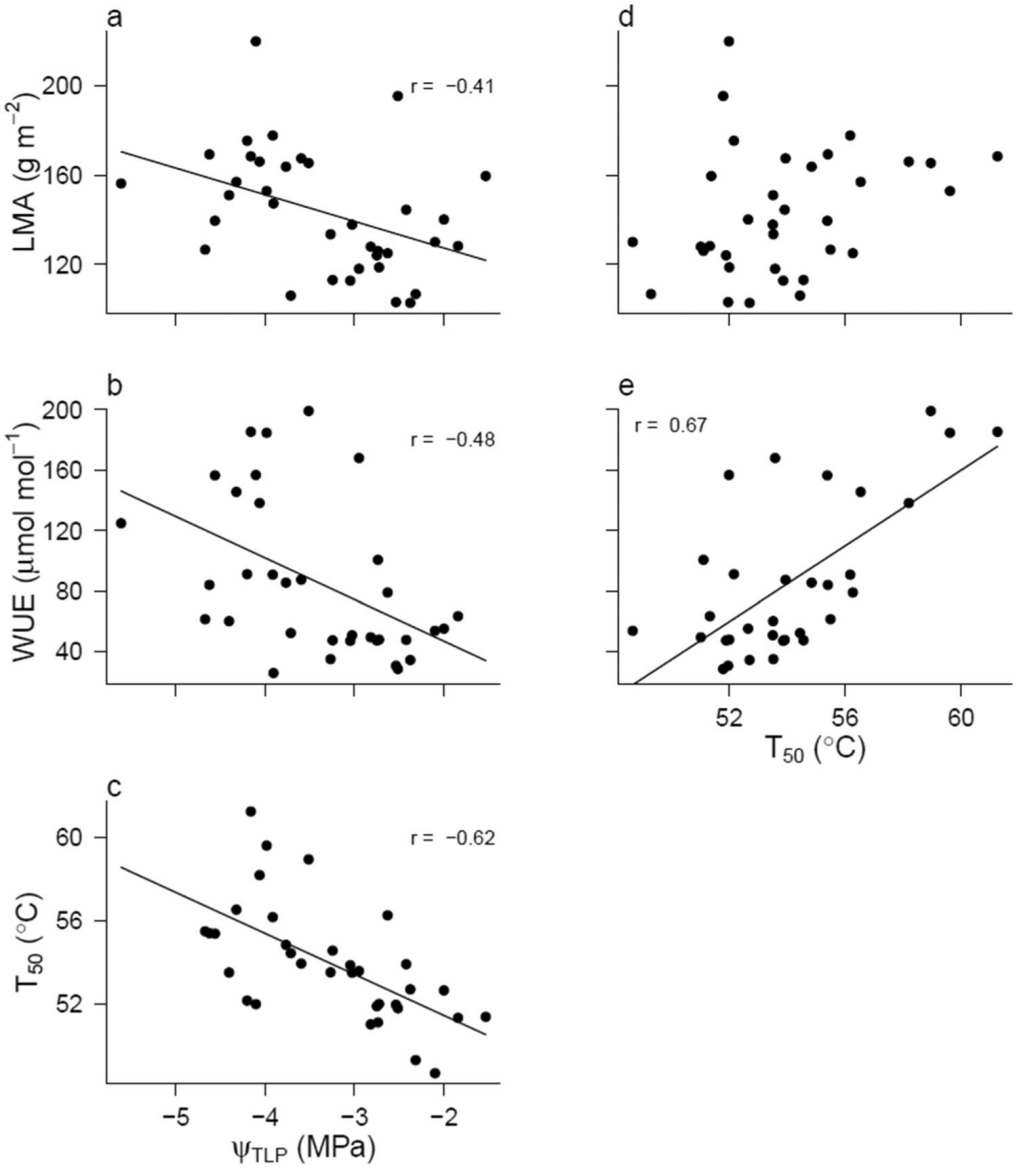
Correlations between physiological parameters. Turgor loss point versus LMA (a), WUE (b) and *T*_50_ (c); *T*_50_ versus LMA (d) and WUE (e). All data was collected at the end of the experiment, in July 2022. Each point represents one individual (*n* = 6 for 6 different species, with drought and well‐watered plants all shown). *R* values indicate the correlation coefficient. A line and correlation coefficient shown indicate a significant correlation between the two variables.

Stomatal closure always occurred at a lower *Ψ* than *Ψ*_TLP_ (Figure 3). The timing of stomatal closure was, however, not uniform, and the MPa difference between stomatal closure and *Ψ*_TLP_ increased with lower *Ψ*_TLP_. A less negative Ψ_TLP_ thus leads to immediate stomatal closure when *Ψ*_TLP_ is reached, while more negative *Ψ*_TLP_ allows for stomatal closure at a lower *Ψ*_leaf_ than *Ψ*_TLP_. *Ψ*_TLP_ was strongly and negatively correlated with LMA, *T*_50_ and iWUE, and *T*_50_ was positively correlated to iWUE (Figure 4, Table S4). This indicates that drought‐tolerant leaves are more heat tolerant, as well as smaller and tougher.

### 3.4 Histological and Cellular Changes After Turgor Loss

**Figure 5:**
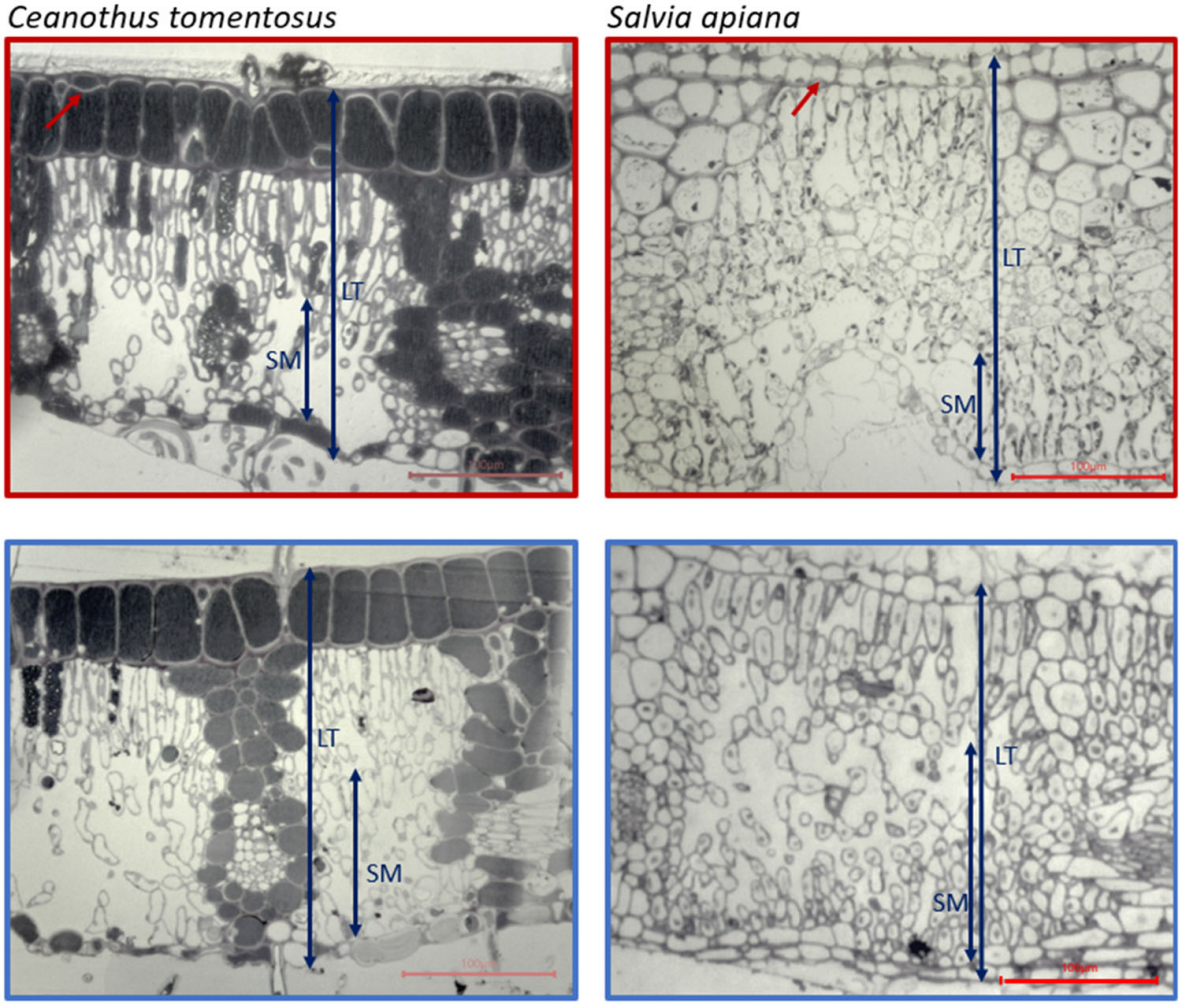
Light microscopy images from *C. tomentosus* and *S. apiana* (cross‐section). Images are taken at 10× magnification with a Leica microscope and camera using AmScope imaging software. Red scale bars indicate 100 μm. Red arrows indicate partial plasmolysis of the epidermal cell. Blue arrows show the parameters measured: LT = leaf thickness, SM = spongy mesophyll thickness. Upper images show drought‐stressed leaves, and lower images show well‐watered leaves. [Color figure can be viewed at wileyonlinelibrary.com]

**Figure 6:**
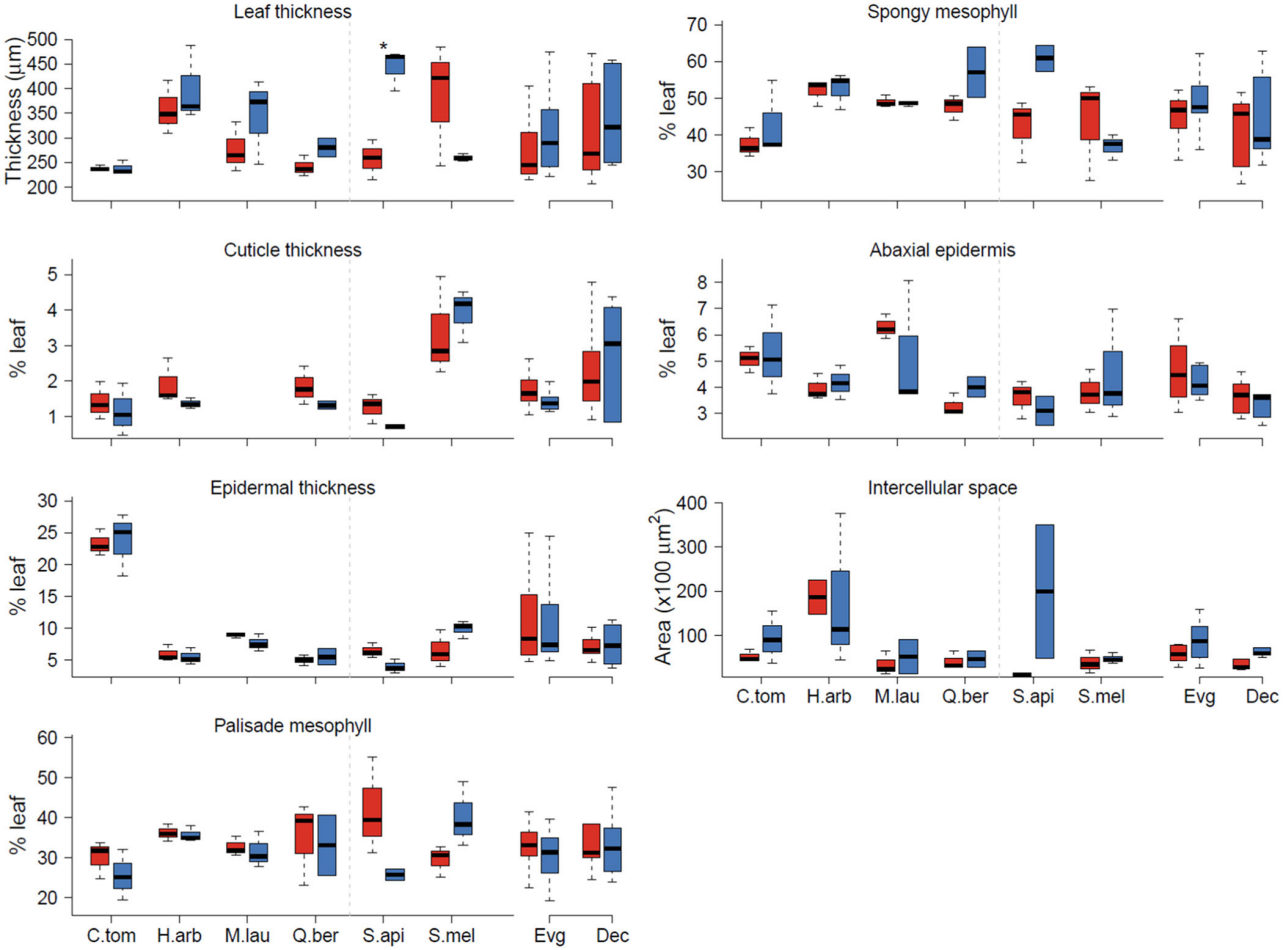
Histological parameters in well‐watered (blue) and drought‐stressed (red) plants of the six species. Boxes show the 95% quantiles. An average of evergreen (*C. tomentosus, H. arbutifolia, M. laurina* and *Q. berberidifolia*) and drought‐deciduous species (*S. apiana* and *S. mellifera*) is given in the right panel for every parameter. Red colours indicate drought‐stressed, and blue colours well‐watered plants. Most parameters are shown as % of leaf thickness. Asterisks indicate a significant difference between well‐watered and dry treatments (*n* = 3). [Color figure can be viewed at wileyonlinelibrary.com]

Signs of plasmolysis were only observed in the epidermal cells of *C. tomentosus* and *S. apiana* (Figure 5). Leaf thickness decreased for all species, though not significant, except for *C. tomentosus* and *S. mellifera* (Figure 6, ns). This reduction was mostly caused by a reduction in the spongy mesophyll and the intercellular air spaces (Figure 6, ns). There was a tendency towards a thicker cuticle in drought‐stressed plants, mainly in the evergreen species (Figure 6, ns).

### 3.5 Correlation Between Drought Tolerance and Histology

**Figure 7:**
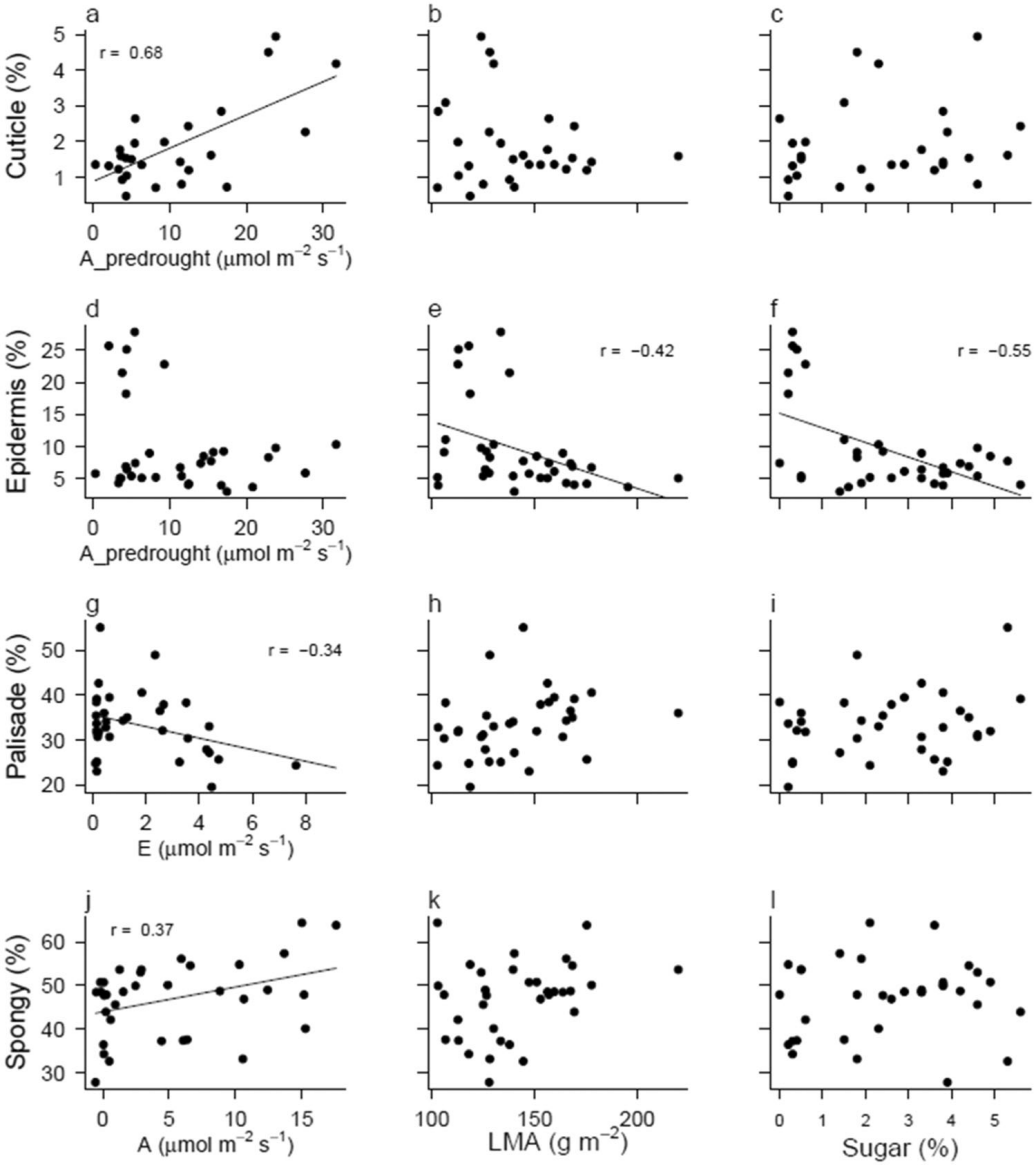
Correlations between histological parameters and physiological traits. Cuticle (a–c), epidermis (d–f), palisade (g–i) and spongy (j–l) mesophyll thickness are expressed in % of leaf thickness and presented in relation to gas exchange rates (a, d, g, j), leaf mass per area (LMA; b, e, h, k) and leaf sugar concentrations (c, f, i, l). Different variants of gas exchange were shown, depending on the statistical significance of the relationship. A_predrought indicates photosynthesis rates before the drought treatment started (March 2022). E and A indicate transpiration and photosynthesis, respectively, at the end of the treatment period (July 2022). Each point represents one individual (*n* = 6 for 6 different species, with drought and well‐watered plants all shown). Data come from measurements taken at the end of the experiment, in July 2022, unless stated differently (‘_predrought’ suffix, April 2022). Lines and correlation coefficients (*r* values) indicate significant correlations between two variables.

Several histological parameters were correlated to physiological traits (Figures 7 and S6, Table S5). A thicker cuticle was correlated to higher pre‐drought photosynthesis levels. The thickness of the epidermis was negatively correlated with LMA and with sugar levels in the leaves. A thicker palisade mesophyll layer was correlated with lower transpiration rates, while thicker spongy mesophyll was positively correlated to photosynthesis rates.

## 4 Discussion

In this study, we connected leaf physiology with cellular structure, to better understand the mechanisms and consequences of extended turgor loss in six native Californian chaparral species. We found that the seasonal minimum (*Ψ*_min_) of three of these species was several megapascals below *Ψ*_TLP_, without signs of leaf mortality or branch dieback (Figure 1). In controlled drought conditions, we found a variation of *Ψ*_TLP_ within species over time as affected by drought and/or season, with no clear distinction between evergreen and deciduous species, confirming our first hypothesis for a part of our species (Figure 2). We found a strong correlation between *Ψ*_TLP_ and thermal tolerance (Figure 4), indicating that drought and heat tolerance are connected, regardless of the many different drought tolerance strategies that can be found within one ecosystem, confirming our second hypothesis. Stomatal closure always happened after turgor loss, but the timing differed between species (Figure 3). We showed that bulk turgor loss did not lead to significant cell plasmolysis or damage but rather to the reduction of leaf thickness, although this was only statistically confirmed in one species (Figure 4). We thus find signs but no strong evidence to confirm Hypothesis 3. Species with low *Ψ*_TLP_ had low photosynthesis and transpiration rates, and high water use efficiency, corresponding to a conservative strategy, while species with a less negative *Ψ*_TLP_ had higher gas exchange rates but also a narrow margin between *Ψ*_TLP_ and stomatal closure (Figure 3). The narrow margin between turgor loss, stomatal closure and cell plasmolysis goes together with a strategy of deciduousness when structural damage is at risk. We discuss these and other correlations from the perspective of leaf structure and function in an extreme drought‐exposed habitat.

### 4.1 Plasticity in *Ψ*_TLP_ and Its Relationship to *T*_50_

We hypothesized that chaparral and coastal sage scrub species would have the ability to adjust their *Ψ*_TLP_ in response to drought (H1). Indeed, we found variable *Ψ*_TLP_ within species (Figure 2). Although only significant for *S. mellifera*, variable *Ψ*_TLP_ was also observed in *M. laurina* and *Q. berberidifolia*. Osmotic adjustment is often speculated to be the mechanism behind *Ψ*_TLP_ adjustment, with sugars as the dominant osmotically active compound (Turner 2018). Here, only the decrease in *Ψ*_TLP_ for *S. mellifera* could be explained by a steep increase in sugar concentrations in its leaves (Figure S5), suggesting that other compounds may have been responsible for a reduction in *Ψ*_TLP_ in the other species. Indeed, often it is the combination of multiple solutes that causes a reduction in *Ψ*_TLP_ (Sanders and Arndt 2012)_._

In *Q. berberidifolia*, a reduction in *Ψ*_TLP_ of more than 2 MPa was observed. These extreme changes observed confirm findings in the literature on the high plasticity of *Quercus* spp. (García‐Plazaola et al. 1999; Schönbeck et al. 2022). However, the magnitude of the changes is remarkable and points to a more careful interpretation of global or average values of *Ψ*_TLP_ in research. On a global scale, the variation range of *Ψ*_TLP_ can probably be averaged over all species (Bartlett et al. 2014), but global published data of *Ψ*_TLP_ should not be used to describe the drought tolerance of a species within their ecosystem over time, or between ecosystems, as variation can lead to large errors. Therefore, we argue that the reference point should be considered before assessing the status of an individual or species in its habitat (Schönbeck et al. 2023).

Interestingly, in the case of *Q. berberidifolia*, *Ψ*_TLP_ did change in response not only to drought but also to other seasonal changes, in well‐watered plants. This would indicate that other factors, such as temperature, might induce a programmed adjustment of *Ψ*_TLP_. The physiological mechanisms behind temperature and drought acclimation and their respective roles are still largely unknown. Our hypothesis that *Ψ*_TLP_ and *T*_50_ would be related was confirmed (H2, Figure 4), indicating a coordinated leaf response to heat and drought that has been shown to hold both within and across ecosystems (Mitchell 2021; Münchinger et al. 2023).

### 4.2 Absence of Cell Plasmolysis Beyond Turgor Loss, While Stomatal Conductance Is Maintained

Stomatal closure (gs90) always occurred below *Ψ*_TLP_. *Ψ*_TLP_ and gs90 were strongly correlated but not at a 1:1 ratio (Figure 3). Species with more negative *Ψ*_TLP_ had a larger margin towards stomatal closure. While it is generally assumed that stomatal closure occurs soon after *Ψ*_TLP_ due to loss of turgor in stomatal guard cells (Bartlett et al. 2016), or even above *Ψ*_TLP_ due to ABA signalling to prevent wilting (Hsu et al. 2021), we show here that this theory does not hold for the drought‐tolerant species measured in this study. Similar findings were reported by Farrell et al. (2017), who compared drought avoiders and drought‐tolerant dryland plants and found that drought‐tolerant plants were able to keep their stomata open at lower water potentials than their *Ψ*_TLP_.

In many species and ecosystems, minimum *Ψ*_leaf_ at midday (*Ψ*_min,MD_) does not reach lower than *Ψ*_TLP_ (Bartlett et al. 2016). In our study, we show how Mediterranean species show midday *Ψ*_leaf_ several megapascals below their *Ψ*_TLP_ at the end of a dry season (Figure 1), with continued stomatal conductance (Figure 3). These results raise the question about the physiological consequences of maintaining living tissue beyond turgor loss. We hypothesized that *Ψ*_TLP_ would result in selective cell plasmolysis while conserving photosynthetic function (H3). We found that none of the species studied showed extensive cellular damage or plasmolysis below *Ψ*_TLP_ (Figure 5). The sturdy xerophyllous leaves most likely provide a strong matrix of support that prevents leaves from wilting. Only in two species—*C. tomentosus* and *S. mellifera*—some plasmolysis of epidermal cells was visible (Figure 5), while the photosynthetically active mesophyll cells remained intact. Other studies confirm that delaying drought damage of mesophyll cells may be a successful strategy to stay photosynthetically active (Azzarà et al. 2023). Similar protection mechanisms were observed—albeit at *Ψ*_leaf_ values above *Ψ*_TLP_—during summer drought in invasive species in the Mediterranean basin (Azzarà et al. 2023). This mechanism would require an uneven distribution of *Ψ* throughout the leaf, with airspaces getting unsaturated due to water moving to the mesophyll to support photosynthesis. Such airspace unsaturation is not detectable with bulk measurement of *Ψ*_TLP_, and currently, leaf models do not assume uneven *Ψ* across leaf tissues (Scoffoni et al. 2023). These and other recent findings might thus implicate an updated requirement of leaf hydraulic models.

### 4.3 Sturdiness Versus Deciduousness

In line with, for example, Scoffoni et al. (2014), low *Ψ*_TLP_ was correlated to high LMA and water use efficiency (Figure 4). Low *Ψ*_TLP_ was also correlated to lower transpiration and assimilation rates. Overall, we find a large gradient of strategies within one ecosystem with ‘drought‐tolerant species’, a large gradient of strategies can be found. Both relatively high‐risk, acquisitive species—with less negative *Ψ*_TLP_ and high assimilation rates—and species with more conservative strategies are present in the Californian chaparral. High risk goes hand in hand with (semi‐)drought‐deciduousness, where fresh foliage replaces old foliage during the wetter winter months. *S. mellifera, S. apiana* and *C. tomentosus* belong to those ‘high risk’ groups.

An interesting correlation observed was the positive correlation between cuticle thickness and photosynthesis rates. The cuticle is, together with cuticular hairs, a protective barrier that increases the boundary layer resistance and protects the leaf from outside stressors (Yeats and Rose 2013; Schuster et al. 2016). The thicker cuticle corresponding to higher assimilation and transpiration rates (Figure 7, Table S5) seems to support the theory that the thickness of the cuticle does not define the quality of the barrier against transpiration (Schuster et al. 2016). Among others, *S. mellifera* belongs to the species with a thicker cuticle. This species apparently reaches high photosynthesis rates by having dense spongy mesophyll with little airspaces that increase the efficiency of gas movement between the cells.

The negative relationship between intercellular space (in 2D) and photosynthesis (*A*) suggests that smaller air spaces, and thus denser mesophyll, increase the level of photosynthesis. In a study with evergreen oaks, this observation was interpreted as a strategy to reach high *A* despite higher LMA (Peguero‐Pina et al. 2017). The results here corroborate this hypothesis.

## 5 Conclusions

In this study, we show the connection between leaf structure and function, by connecting physiological measurements with leaf histology measurements in six native chaparral species during drought stress. We showed that the assumed bulk leaf *Ψ*_TLP_ does not lead to significant cell plasmolysis in the most drought‐tolerant species. This goes together with the fact that these species are able to keep their stomata open and transpire after *Ψ*_leaf_ falls below turgor loss. Furthermore, we found an intraspecific variation of *Ψ*_TLP_ and *T*_50_ that is not only drought driven but potentially also temperature driven. The acclimation patterns to drought and heat, especially their individual roles, have to be identified more closely for each species to fully understand their resilience to climate change.

## Conflicts of Interest

The authors declare no conflicts of interest.

## Supporting information

Schonbeck etal SupportingInformation.docx.

## Data Availability

The data that support the findings of this study are available from the corresponding author upon reasonable request.
