# Supplementary material for: Leaf Turgor Loss Does Not Coincide With Cell Plasmolysis in Drought‐Tolerant Chaparral Species
Source: Plant Cell Environ. 2025 Mar 27;48(7):5019–29. doi: 10.1111/pce.15505 (PMC12131956; doi:10.1111/pce.15505)
Supplement: Supplementary file 1 — Schonbeck etal SupportingInformation.docx. [file PCE-48-5019-s001.docx]

Supporting information

**Title:** Leaf turgor loss does not coincide with cell plasmolysis in drought tolerant Chaparral species

**Authors:** Leonie C. Schönbeck^1,2*^, Carolyn Rasmussen^1^, Louis S. Santiago^1,3^

^1^ University of California, Riverside, Department of Botany & Plant Sciences, Riverside CA 92521 USA

^2^ Swedish University of Agricultural Sciences, Southern Swedish Forest Research Center, Alnarp, Sweden

^3^ Smithsonian Tropical Research Institute, Apartado 0843-03092. Balboa, Ancon, Panama

* Corresponding author: Leonie Schönbeck

Southern Swedish Forest Research center Swedish University of Agricultural Sciences Sundsvägen 3

PO box 190, SE-234 22 Lomma [Leonie.schonbeck@slu.se](mailto:Leonie.schonbeck@slu.se)

# The following supplementary data is available:

Table S1: Leaf traits of the six studied species

Table S2: Anova table of turgor loss point against time and treatment effect

Table S3: Anova table of treatment differences for total non-structural carbohydrates and sugars Table S4: Correlations between physiological leaf traits.

Table S5: Correlations between histological and physiological leaf traits.

Figure S1: Temperature, humidity, VPD and soil VWC during the drought experiment Figure S2: Midday leaf water potential in well-watered and drought-stressed plants. Figure S3: Relationship between published and measured ΨTLP values

Figure S4: Photosynthesis measured biweekly in well-watered and drought-stressed plants. Figure S5: Non-structural carbohydrates in leaves of the six study species

Figure S6: Correlation plots of each measured variable

**Table S1.** Leaf traits of the six studied species. LMA and Amax are taken from own measurements. TLP range is shown for literature values (lit) and those that were measured in this study (measured). Estimated rooting depth is taken from (Pivovaroff, Cook & Santiago 2018)

| **Species** | **Deciduousness** | **LMA**  **(g m-2)** | **Amax**  **(µmol m^-2^ s^-1^)** | **TLP range lit**  **(MPa)** | TLP measured | **Est. rooting**  **depth (m)** |
| --- | --- | --- | --- | --- | --- | --- |
| *C. tomentosus* | Semi-deciduous | 49 ± 0.006 | 4.89 ± 0.99 | -3.08 – -2.63 | -3.08 – -2.72 | 1.8-3.7 |
| *H. arbutifolia* | Evergreen | 284 ± 0.018 | 4.69 ± 0.47 | -4.33 – -3.65 | -4.56 – -3.51 | 0.3 |
| *M. laurina* | Evergreen | 221 ± 0.018 | 12.17 ± 2.06 | -4.28 – -3.35 | -4.67 – -3.01 | 13.2; 5.4 |
| *Q. berberidifolia* | Evergreen | 66 ± 0.014 | 7.89 ± 2.09 | -4.72 – -2.59 | -5.21 – -3.9 | 10.7 |
| *S. apiana* | Deciduous | 172 ± 0.028 | 14.69 ± 2.21 | -2.29 – -1.97 | -2.63 – -1.95 | 2.7; 1.5 |
| *S. mellifera* | Deciduous | 76 ± 0.006 | 24.56 ± 2.50 | -2.70 – -2.08 | -2.82 – -1.84 | 2.1 |

**Table S2.** Anova table of turgor loss point against time and treatment effect for each of six species studied (n=3). Bold characters indicate significant (p < 0.05), italics characters indicate marginally significant effects.

|  | *F* | *p* |
| --- | --- | --- |
| *C. tomentosus* |  |  |
| Time | 2.26 | 0.171 |
| Treatment | 0.61 | 0.456 |
| Time * Treatment | 1.08 | 0.328 |
| *H. arbutifolia* |  |  |
| Time | 0.03 | 0.873 |
| Treatment | 2.43 | 0.157 |
| Time * Treatment | 0.87 | 0.378 |
| *M. laurina* |  |  |
| Time | 2.08 | 0.223 |
| Treatment | 2.11 | 0.220 |
| Time * Treatment | *7.62* | *0.051* |
| *Q. berberidifolia* |  |  |
| Time | **23.40** | **0.001** |
| Treatment | 1.70 | 0.229 |
| Time * Treatment | 0.21 | 0.660 |
| *S. apiana* |  |  |
| Time | 2.78 | 0.171 |
| Treatment | 2.41 | 0.195 |
| Time * Treatment | 6.59 | 0.062 |
| *S. mellifera* |  |  |
| Time | 2.30 | 0.204 |
| Treatment | 3.17 | 0.149 |
| Time * Treatment | 3.91 | 0.119 |

**Table S3.** Anova table for treatment differences in total non-structural carbohydrates and sugar concentrations in leaves. Bold characters indicate significant effects.

|  | **Sugar**  *F* | *p* | NSC  *F* | *p* |
| --- | --- | --- | --- | --- |
| **Treatment** | **8.18** | **0.008** | 2.44 | 0.131 |
| **Species** | **36.11** | **<0.001** | **3.77** | **0.012** |
| **Treatment * Species** | **5.08** | **0.002** | **4.19** | **0.007** |

**Table S4.** Correlations between physiological leaf traits. Only correlation coefficients that were significant (p < 0.05) are given. Apre and Epre indicate pre-treatment levels of gas exchange parameters. ΨTLP = turgor loss point, T50 = temperature at 50% reduction of photosynthetic capacity, LMA = leaf mass per area, Starch = starch concentration in leaves, WUE = water use efficiency, A = photosynthesis, E = evaporation.

|  | **ΨTLP** | **T50** | **LMA** | **Starch** |
| --- | --- | --- | --- | --- |
| **WUE** | -0.482 | 0.665 | 0.500 | 0.371 |
| **Apre** | 0.451 | -0.534 | -- | -- |
| **Epre** | 0.510 | -0.612 | -- | -- |
| **T50** | -0.618 | -- | 0.416 | 0.372 |
| LMA | -0.41 |  |  |  |

**Table S5**. Correlations between histological and physiological leaf traits. Only correlation coefficients that were significant (p < 0.05) are given. If value is accompanied with “pre”, the pre-treatment levels of gas exchange are meant. Otherwise, gas exchange levels during the experiment are used.

|  | **LMA** | **A** | **E** | **WUE** | **Sugar** | **T50** |
| --- | --- | --- | --- | --- | --- | --- |
| **Cuticle** | -- | 0.681 pre | 0.666 pre | -- | -- | -0.395 |
| **Epidermis** | -0.415 | -- | -- | -- | -0.554 | -- |
| **Palisade** | -- | -- | -0.340 | -- | -- | -- |
| **Spongy** | -- | 0.372 | 0.343 | 0.365 | -- | -- |
| **Intercell. Space** | -- | -0.381 pre | -0.376 pre | -- | -0.395 | -- |


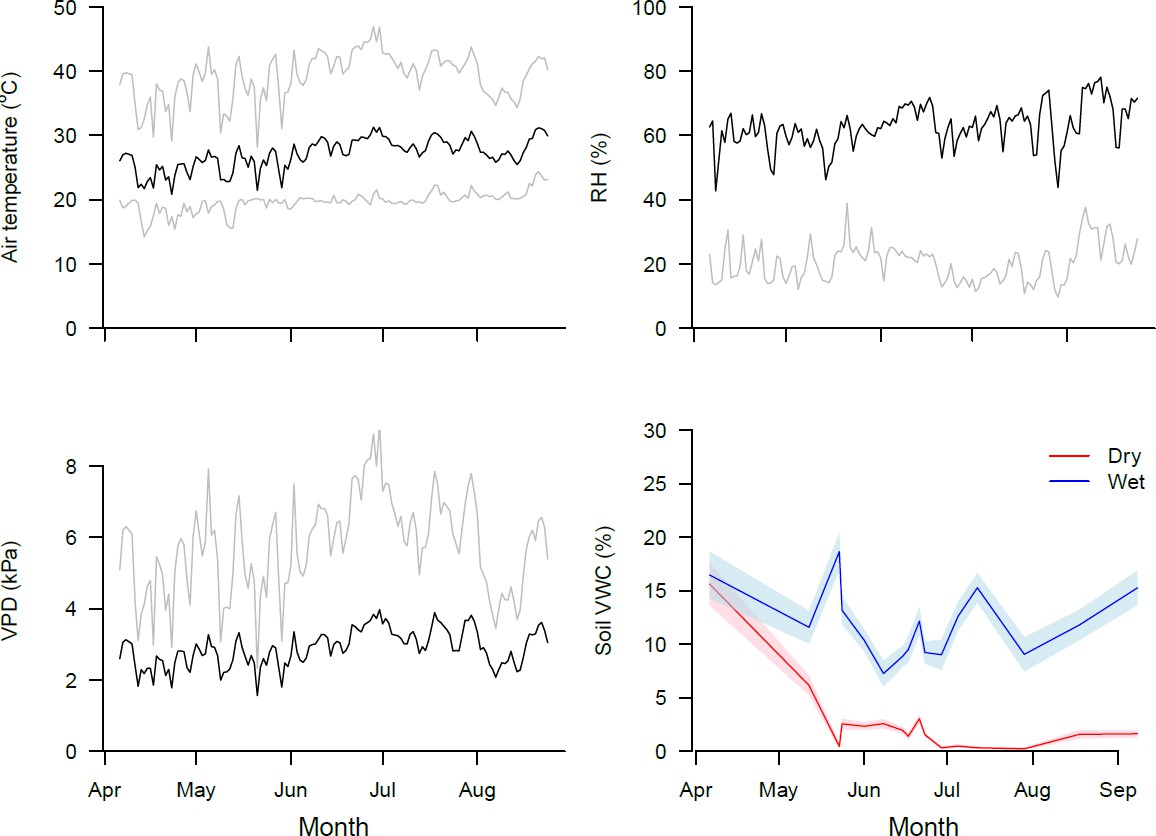


**Figure S1.** Temperature, humidity, VPD and soil VWC during the drought experiment. VWC shows average treatment values for all species, with bands showing SE.


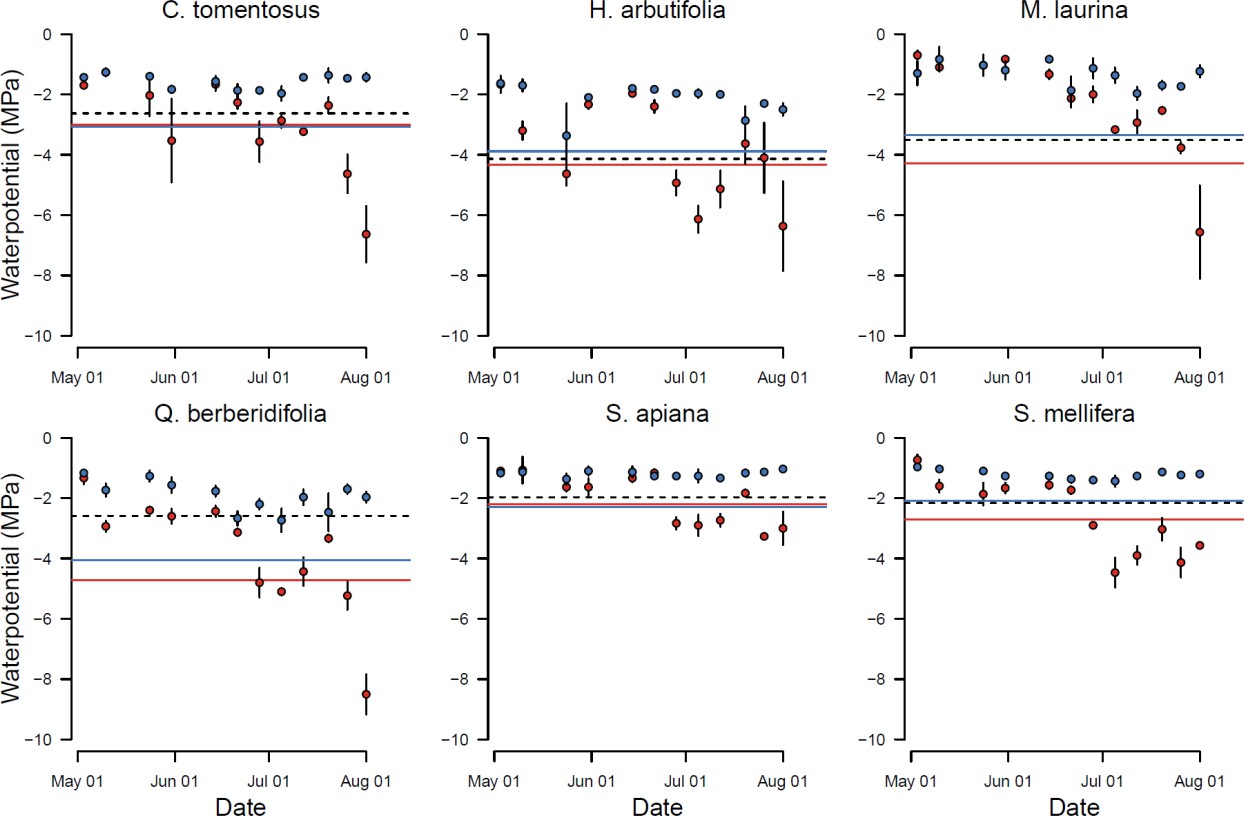


**Figure S2.** Midday leaf water potential in the six species in well-watered (blue) and drought-stressed (red) plants. Dashed lines shows pre-treatment turgor loss point for the species, as measured in April 2022. Blue and red lines show the turgor loss point in well-watered (blue) and drought-stressed (red) plants at the end of the treatment period, in August 2022. Error bars indicate SE (n=3)


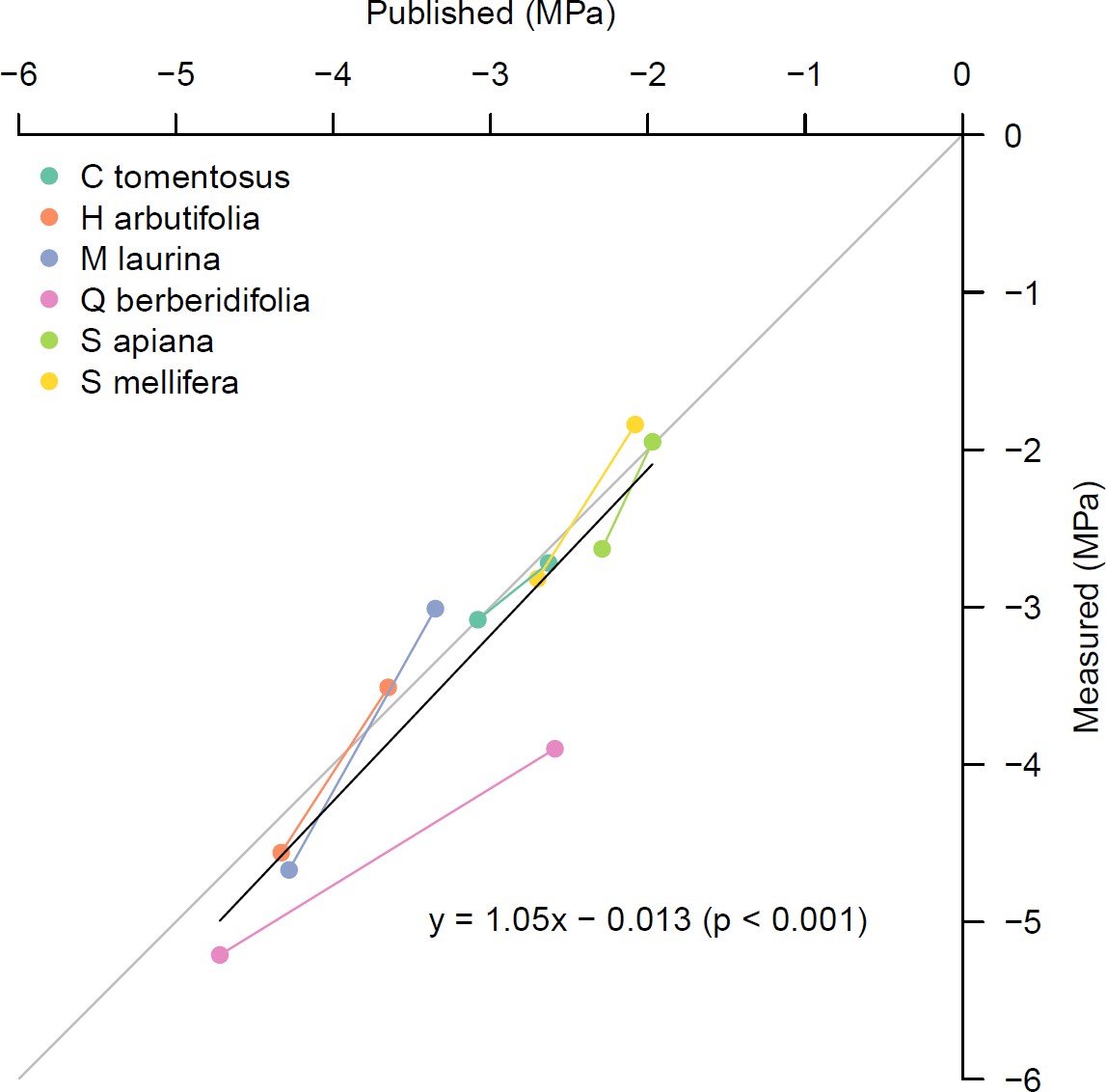


**Figure S3.** The minimum and maximum values of published (x-axis) versus measured (y-axis) ΨTLP values.

The majority of the published values originate from PV-curves, while the measured values come from osmometer measurements. The close to 1:1 relationship assumes no issues with rehydration or

differences in methodology.


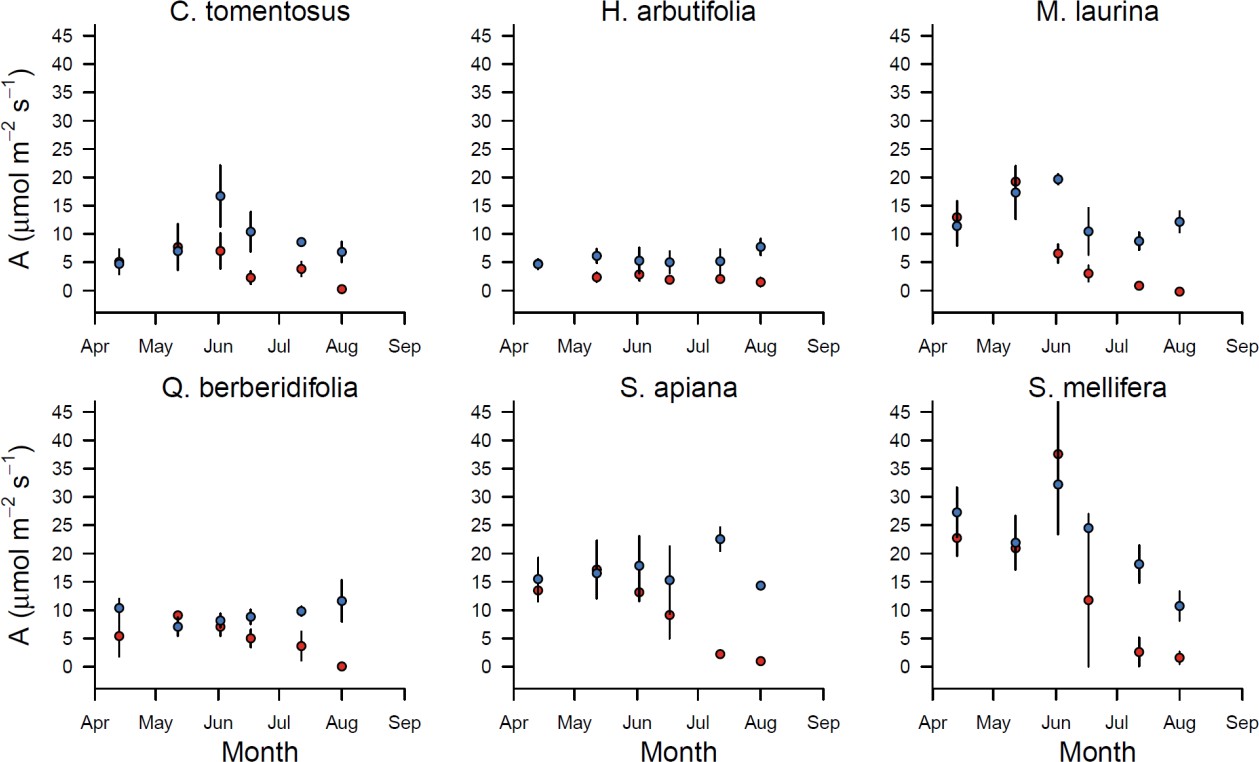


**Figure S4.** Photosynthesis measured biweekly in well-watered (blue) and drought-stressed (red) plants of the six study species. Error bars indicate SE (n=3).


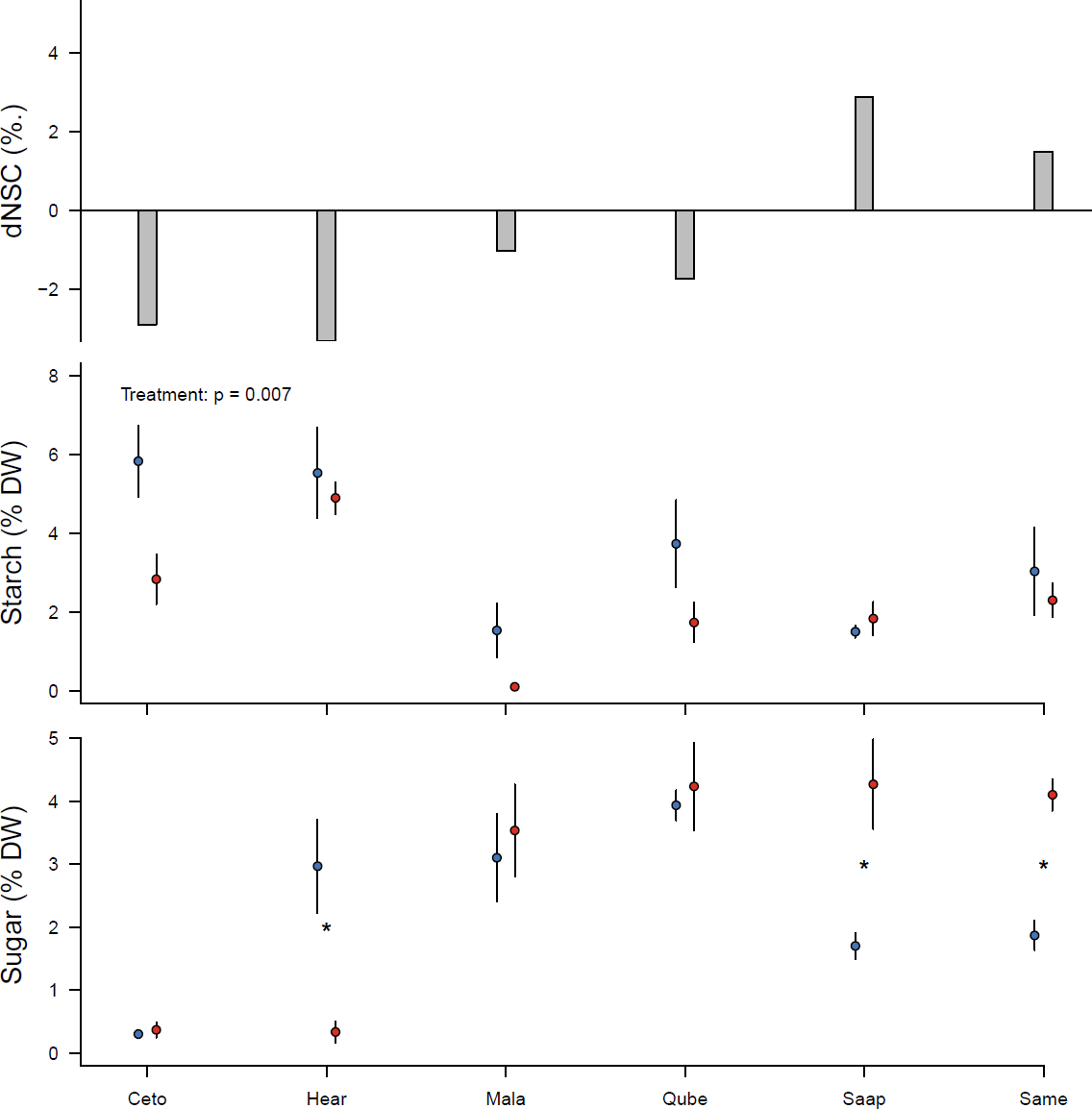


**Figure S5.** Non-structural carbohydrates in leaves of the six study species in August 2022. Upper panel: difference between total NSC in well-watered and drought-stressed plants (negative symbols indicate

lower NSC in dry plants); middle panel: Percentage starch in the leaves; Lower panel: percentage of sugar in leaves. Blue symbols indicate well-watered, red symbols drought-stressed plants. Segments indicate SE. Asterisks indicate significant difference within a species between well-watered and drought-stressed plants.


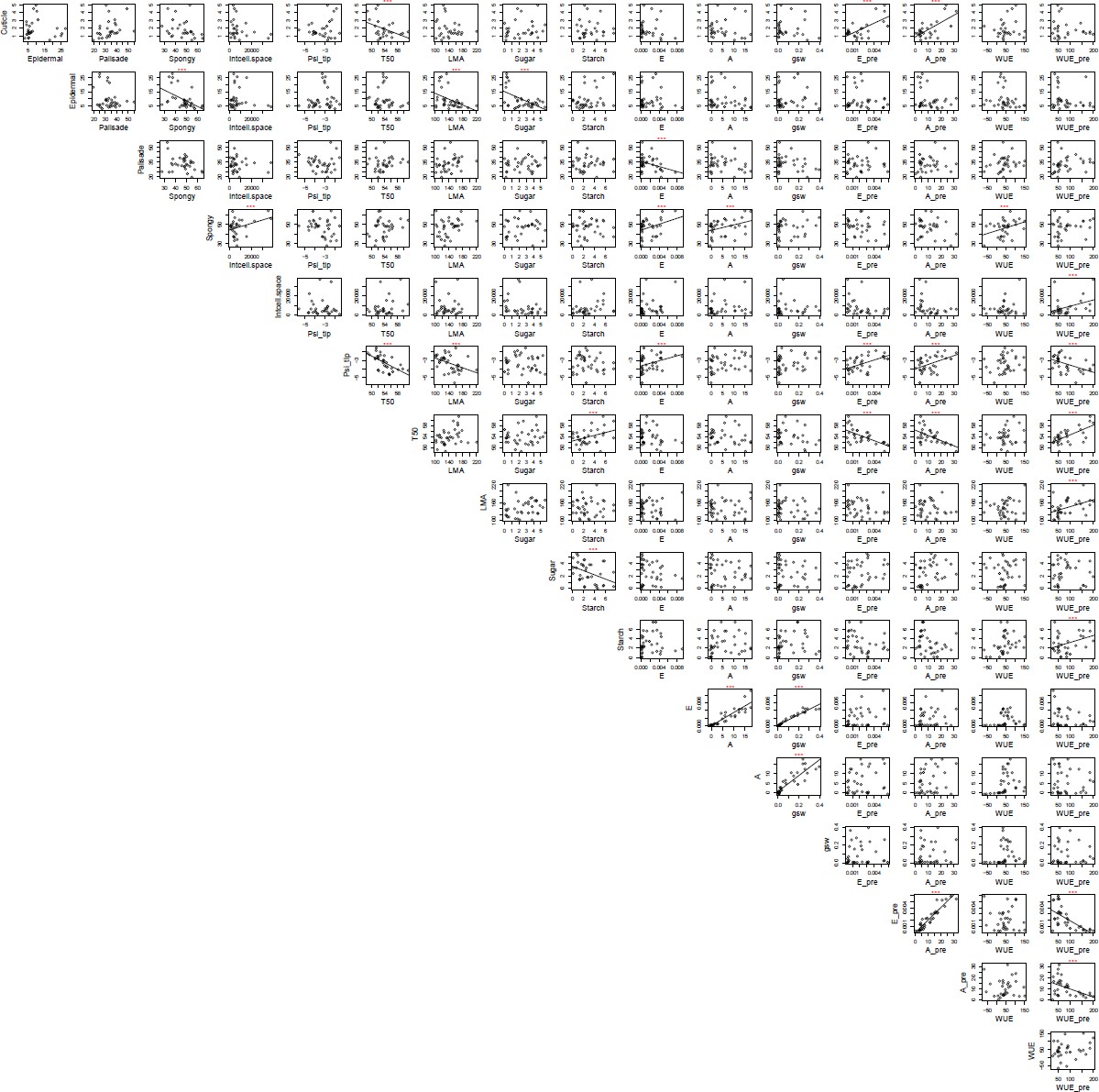


**Figure S6.** Correlation plots of each individual measured variable. Red asterisks above the plot indicate significant correlation.

# References

Pivovaroff A.L., Cook V.M.W. & Santiago L.S. (2018) Stomatal behaviour and stem xylem traits are coordinated for woody plant species under exceptional drought conditions. *Plant, Cell & Environment* **41**, 2617–2626.
